# Exosomes as New Generation Vehicles for Drug Delivery: Biomedical Applications and Future Perspectives

**DOI:** 10.3390/molecules27217289

**Published:** 2022-10-27

**Authors:** Amarjitsing Rajput, Akansh Varshney, Rashi Bajaj, Varsha Pokharkar

**Affiliations:** Department of Pharmaceutics, Poona College of Pharmacy, Bharti Vidyapeeth Deemed University, Erandwane, Pune 411038, Maharashtra, India

**Keywords:** exosomes, biogenesis, isolation, drug delivery, therapeutic applications

## Abstract

Currently, particular interest among the scientific community is focused on exploring the use of exosomes for several pharmaceutical and biomedical applications. This is due to the identification of the role of exosomes as an excellent intercellular communicator by delivering the requisite cargo comprising of functional proteins, metabolites and nucleic acids. Exosomes are the smallest extracellular vesicles (EV) with sizes ranging from 30–100 nm and are derived from endosomes. Exosomes have similar surface morphology to cells and act as a signal transduction channel between cells. They encompass different biomolecules, such as proteins, nucleic acids and lipids, thus rendering them naturally as an attractive drug delivery vehicle. Like the other advanced drug delivery systems, such as polymeric nanoparticles and liposomes to encapsulate drug substances, exosomes also gained much attention in enhancing therapeutic activity. Exosomes present many advantages, such as compatibility with living tissues, low toxicity, extended blood circulation, capability to pass contents from one cell to another, non-immunogenic and special targeting of various cells, making them an excellent therapeutic carrier. Exosome-based molecules for drug delivery are still in the early stages of research and clinical trials. The problems and clinical transition issues related to exosome-based drugs need to be overcome using advanced tools for better understanding and systemic evaluation of exosomes. In this current review, we summarize the most up-to-date knowledge about the complex biological journey of exosomes from biogenesis and secretion, isolation techniques, characterization, loading methods, pharmaceutical and therapeutic applications, challenges and future perspectives of exosomes.

## 1. Introduction

### 1.1. Background

Description of tiny, secreted vesicles were initially described in the late 1960s by Bonucci and Anderson, who discovered that chondrocytes produce small vesicles of less than 100 nm [1,2]. Furthermore, it was evident from these early investigations that these vesicles were the catalysts for creating hydroxyapatite crystals, budding directly from the plasma membrane [1]. At the same time, Wolf discovered that platelets produce small extracellular vesicles (EVs), often known as platelet dust, which have substantial clotting activity [3]. Platelet dust was found to play a role in thrombin production, which increased the coagulant activity of citrated plasma as storage duration increased [4]. These concurrent, important discoveries have been expanded and verified throughout time, showing functions for osteogenic exosomes in cardiovascular disease-related pathological calcification of arteries and cardiac tissue in addition to normal bone and tooth deposition [5,6]. Extracellular vesicles (EVs) were largely ignored until the 1980s, when EV secretion was recognized as a common cell activity [7]. The extra work on exosomes started in 1980, with ectoenzyme work by Trams et al. [7]. In addition, the exosomes were discovered in the seminal fluid, which was said to originate from the prostate and have the function of transferring proteins and lipids for sperm maturation [8,9]. Thus, EVs are micro/nano-dimension membrane vesicles secreted by all types of prokaryotic and prokaryotic cells [10,11]. Depending on their morphological features and mechanism of formation, EVs have been classified as exosomes (30–150 nm), microvesicles (MVs) (100–1000 nm) and apoptotic bodies (>1000 nm) [12,13,14]. EVs have different components that facilitate cell-to-cell communication with adjacent cells and affect many cellular functions [15,16]. 

The biogenesis mechanism distinguishes exosomes, microvesicles, and apoptotic bodies [17]. Exosomes are of endocytic origin, MVs are produced by budding and blebbing from the plasma membrane, and apoptotic bodies are released by cells undergoing programmed cell death and signal cell engulfment. Exosomes are also named nanosomes due to their size, which ranges from 30 to 150 nm. They can deliver and carry intracellular information and skills by mixing with cells that receive it. As a result, they represent a viable method for developing drug delivery systems that transport a payload to specific cells, tissues, and organs [18]. They are primarily found in several biological fluids, such as urine, saliva, serum, and cerebrospinal fluid (CSF). They are also found in various types of cells like platelets, dendritic cells, B cells and T cells. Earlier studies suggested that exosomes play a crucial role in multiple biological activities such as inflammation, apoptosis, coagulation, intracellular signaling, antigen presentation and cellular homeostasis [19]. Exosomes are small dimension nanovesicles comprising a lipid bilayer, RNAs, and proteins that actively participate in cellular communication. They may be utilized as biomarkers and for therapeutic purposes in diseases [20]. 

The endosomal pathway, which starts with the inner budding of the bio-membrane to create the endosome, a membrane-bound collection of cells, produces exosomes [21,22]. EVs convey payloads with biological signals, such as transmembrane proteins and transferrin receptors, from their cellular origins to the external environment [23]. They communicate with the recipient cell and deliver chemical cargo [24]. Exosomes also aid intercellular communication by transporting proteins and RNA across cells and distant organs [25]. Exosomes attach to cell membranes via receptor-ligand interactions and are involved in antigen presentation, cancer growth, and other processes. They bind to the target cell membrane and open up, releasing their surface protein and cytoplasm into the receiving cell. According to several studies, exosomes penetrate cells, discharge their cargo, and influence various physiological and pathological processes [26]. 

During exosome production, inside the lumen or lipid bilayer, biomolecules such as cell-targeting constituents, cell adhesion proteins, coding, and non-coding nucleic acids are enveloped, contributing to the exosome’s multiple features [27]. The exact cellular release site, the EV’s origin, physiological and pathological status, and even the particular cellular release location influence this. Exosomes contain various proteins, including those involved in vesicle formation and trafficking, indicating the presence of disease pathologies such as cancer or inflammatory diseases. However, exosomes also contain many common proteins involved in vesicle formation and trafficking [28] 

It has been identified that exosomes comprise 9769 proteins, 3408 mRNAs, 2838 miRNAs, and 1116 lipids [29,30]. Its constituents, including particular lipids, proteins, DNA, mRNA, and noncoding RNAs, can serve as autocrine and paracrine agents. Exosome contents can be utilized as prognostic indicators and a grading system for cancer development. It also controls tumor development, metastasis, and angiogenesis in tumor cells and mediates treatment resistance [31]. Exosomes’ ground-breaking preclinical success as a remedy for these unmet clinical issues has given them a lot of relevance as a potential drug delivery vehicle in recent years [32]. An in-depth understanding of exosome biology is essential to ensure the clinical development of exosome-based therapeutic products.

### 1.2. Structure and Biogenesis

#### 1.2.1. Structure

**Figure 1:**
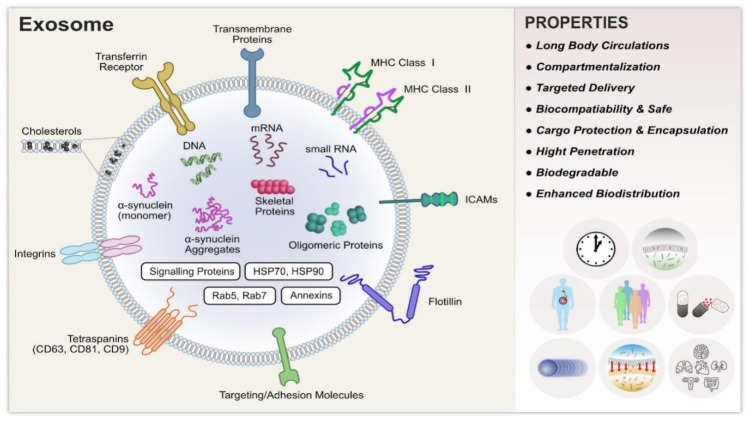
Typical structure of exosomes, properties, and functional attribution of various biomolecules present in exosomes. Adopted with kind permission from reference [35].

Exosomes have a single lipid bilayer membrane in the vesicle, have the same topology as the cell, and are 40–100 nm in diameter. The exosome size varies, even when secreted from a single cell, but all the characterization techniques display spherical morphology [33]. Exosomes have been reported to have a density of 1.1–1.2 g/mL, wherein the density of the vesicle is affected by the protein: lipid ratio, and it varies from vesicle to vesicle [34]. The exosomes have different surface proteins, such as tetraspanins and integrins, performing cell adhesion functions. Due to the resemblance of their structure to the cells, exosomes have high penetration, prolonged blood circulation, biocompatibility and enhanced biodistribution [21]. The basic structure of exosomes is shown in Figure 1, adopted with permission from reference [35]. The composition of exosomes with the functions and examples of the various components are shown in Table 1.

#### 1.2.2. Biogenesis

Exosome biogenesis follows the endosomal trafficking pathway and is related to the endosomal system. The endosomal system comprises early endosomes, multivesicular bodies, and primary endocytic vesicles [36]. 

**Figure 2:**
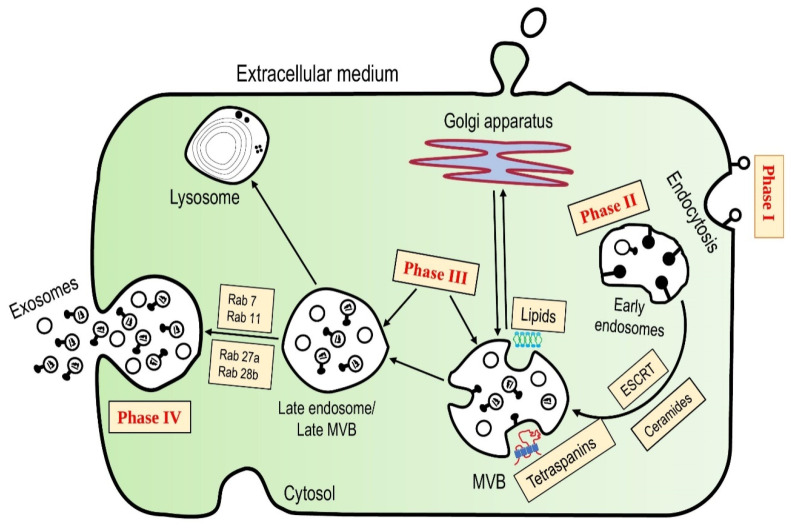
Exosome biogenesis; Phase I—Initiation, Phase II—Endocytosis, Phase III—Multivesicle formation, Phase IV—Secretion of exosomes.

Exosome biogenesis and secretion occur in four phases, as illustrated in Figure 2. 

Phase 1 is initiation, wherein the exosomes are created by the restricted multivesicular body (MVB) membrane budding inward. Removing the plasma membrane (PM) from the cells through inward budding or endocytosis is the first step in creating exosomes. Phase II represents the formation of an early endosome. The lipid raft domains of PM are primarily responsible for forming these endocytic vesicles. This method is so fast that a reticulocyte’s PM can be regenerated in as little as 1 h. Early endosomes are produced intracellularly. Zhang et al. demonstrated the role of the transport-related endosomal sorting complex (ESCRT) in forming ILVs [37]. The complicated protein machinery comprises four different protein ESCRTs (0 through III) that aid MVB production, vesicle budding, and protein cargo sorting [37]. The ESCRT mechanism begins when the ESCRT-0’s ubiquitin-binding subunits recognize ubiquitinated proteins and sequester them to phosphatidylinositol-3-phosphate (PI3P) containing endosomal membrane regions. The PI3P is a type of phospholipid found abundantly in early and late endosomes involved in signal transduction and membrane trafficking [38]. The ESCRT-III complex then uses the energy of the sorting protein Vps4 to detach from the MVB membrane after cleaving the buds to produce ILVs [37].

Cargoes like mRNA and proteins gather inside endosomes later in the cell, resulting in multivesicular endosomes or intraluminal vesicles [35].

Phase III constitutes the Multivesicle body (MVB) and the late endosome formation. This process is governed by several routes, some of which are still unknown. The two types of pathways are the endosomal sorting complexes needed for transport (ESCRT)-dependent and ESCRT-independent mechanisms. ESCRT includes ESCRT-0, ESCRT-I, ESCRT-II, and ESCRT-III—all cytosolic protein complexes. The limiting membrane of endosomes may flex and modify due to the activity of ESCRT and its accessory protein, allowing cargo, such as cytosol, to be transported into the lumen.

To put it another way, early endosomal membrane budding occurs inside. MVBs (multivesicular bodies) are formed as a result of this. The Golgi complex is also involved in this process. The early endosomes are then transformed into late endosomes. Alongside ESCRT-dependent techniques, the roles of complex lipids and other protein-related pathways in exosome generation have also been highlighted. Complex lipids, such as ceramide, can self-associate to form raft-like structures and contribute to the initial membrane curvature for inward budding to form intraluminal vesicles. The ceramides play an essential role in the grouping into the microdomains, one possible strategy for loading the MVB [35]. Furthermore, the importance of mechanisms relying on tetraspanins and lipids cannot be underestimated and has helped improve our understanding of exosome generation and release dynamics.

Phase IV relates to the exosome secretion occurs. The MVBs can have one of two final fates; the exosome can undergo fusion with lysosomes or get secreted as the extracellular discharge of internal cargos into the extracellular area after fusion with PM. Rab27a and Rab27b, two members of the guanosine triphosphatase (GTPase) family, have been shown to help MVBs dock at the PM. Rab27a is said to diminish the size of MVBs, whereas Rab27b is said to aid the MVBs distribution outside the perinuclear area [35].

**Table 1:** Composition of exosomes, functions of components and examples; adapted with kind permission from reference [39].

Composition	Function	Examples
Lipids and Metabolites	Vesicle formation	Glycosphingolipids Monosialotetrahexosyl ganglioside (GM3) Sphingomyelin Cholesterol Phosphatidylserine Prostaglandins Glycerophospholipids organic acids Alcohols, Steroids, Phenols, amino acids conjugates, Sugar and conjugates
	Biogenesis	
	Release & interaction with target cells	
	Pathophysiological conditions	
	Inflammatory processes	
	Rigidity	
Proteins	Transporters	ATP7A, ATP7B, MRP2, SLC1A4, SLC16A1, CLIC1
	Receptors	CD46, CD55
	Heat shock proteins	Hsc70, Hsp70, Hsp90
	Tetraspanins	CD9, CD81, CD82
	Metabolic enzymes	GAPDH, Pyruvate
	Antigen presentation proteins	HLA Class I & II, Peptide complexes
	Lysosomal markers	CD63, Lysosome membrane protein 2
	Membrane adhesion proteins	Integrins
Nucleic Acids	Mediator of horizontal transfer of genetic information	mRNA
	Gene regulation	Non-Coding RNA
	Target cells gene silencing	miRNA
	Carcinogenesis and cancer progression	Long Non-Coding RNAs

### 1.3. Role of Exosomes

EVs were mainly identified in the 1980s (1983) as involved in eliminating cellular waste through the reticulocyte scavenging pathway [40,41]. Nowadays, EVs are established as vital vesicles that enable intercellular communication in coordination with the endocrine and pancreatic systems. Exosomes have been identified to have a plethora of functions; for example, they have been demonstrated to play a critical role in embryonic development [36] and their immunomodulatory action [42,43]. They also promote tissue regeneration and help wound-healing activity [44,45]. EVs enhance viral particle transmission and spread in particular diseases [46,47], aid metastasis and tumorigenesis [48,49] and play an essential role in neurodegenerative diseases [50,51]. EVs are now being researched for their potential to serve as carriers for targeted drug delivery of drugs and biologics to disease tissue cells [52]. 

### 1.4. Types of EVs 

EVs are those that have been isolated from body fluids, serum, and conditioned cell growth medium, which is made up of several varieties of closed membrane vesicles. These vesicles showed a specific change in size, shape, composition and biogenesis process. EVs consist of four subclasses,

(a) Exosomes (30–150 nm);
(b) Oncosomes (100–1000 nm);
(c) Ectoderms/Microvesicles (100–1000 nm);
(d) Apoptotic bodies (200–2000 nm) [51].

Mainly tumor cells or apoptotic cells are responsible for producing oncosomes and apoptotic bodies [52].

## 2. Isolation and Separation Techniques for Exosomes

Exosomes have been separated from various fluids (biological) like cerebrospinal fluid (CSF), saliva, urine, amniotic fluid, semen, breast milk, tears, etc. [53,54]. It can also be separated from conditioned mediums like tissues, cultured organs, and cell lines [55]. In most cases, exosomes are obtained from conditioned medium and biological fluids by suitable laboratory techniques such as ultrafiltration, immunological separation, ultracentrifugation, size exclusion chromatography (SEC), precipitation based on polymer, magnetic separation, acoustic fluidic separation, dielectrophoretic separation, deterministic Lateral Displacement (DLD) separation and currently microfluidic devices [56,57]. The methods used to isolate and separate the exosomes are discussed below.

### 2.1. Ultrafiltration

The separation of exosomes is a simple, size-dependent, and convenient technique [58]. Ultracentrifugation is a slower, more complex, and less productive process. The advantage of this process is that it only takes a small sample to get the required production.

Cheruvanky et al. showed that exosomes could be separated successfully using 0.5 mL of the urine sample by ultrafiltration, indicating its high efficiency [59]. Direct flow filtration and tangential flow filtration techniques were used to perform ultrafiltration. Direct flow filtration is the commonly used technique for small-volume sample filtration (up to 30 mL). But this filtration method suffers from membrane fouling problems and poor particle separation. For large-scale exosome extraction, tangential flow filtration is much more effective and practical in preventing clogging and cake formation; the sample stream runs over the ultrafilter membrane in TFF [60]. 

### 2.2. Immunological Separation

Immunoaffinity is another approach for exosome purification. Affinity purification employing antibodies to CD63, CD81, CD82, CD9, EpCAM, and Rab5 is better for specific exosome isolation. These could be used separately or in combination. Magnetic beads, chromatography matrices, plates, and microfluidic devices could all be used to immobilize antibodies for this application [61,62]. Although this approach is scalable, most extracellular exosome-associated antigens are not specific to exosomes. Protein complexes and other particulates, including antigen-carrying non-exosome vesicles like cell debris, could be purified using immunoaffinity-based isolation techniques. However, this procedure may isolate exosomes exclusive to a particular biomarker [27,63].

### 2.3. Ultracentrifugation

The traditional and gold standard method for isolating exosomes is ultracentrifugation. Based on density, size, and form, this technique uses centrifugal force to remove cells, and the significant cell remains out of biological fluids [35] héry et al. published the following experimental methodology to extract and separate exosomes via ultracentrifugation [62].

Exosome isolation should be improved by ultracentrifugation with density gradient separation as centrifugation technology improves [64]. Although ultracentrifugation is a well-established method for isolating exosomes, prolonged usage can cause the exosome membrane to rupture due to centrifugal force’s effects on the exosome [65].

### 2.4. Size Exclusion Chromatography

The chromatographic technique known as size exclusion chromatography (SEC) traps particles smaller than the pore in a column with porous beads. Particles that are larger than the pore are quickly eluted in contrast. Exosomes have a diameter of 40–100 nm, although most proteins have a diameter of less than 10 nm. Using centrifugation, exosomes can be separated from proteins to remove more significant cell remains, accompanied by beads of suitable size. Exosomes have been extracted from protein using Sepharose CL-4B, Sephacryl S-400, and Sepharose CL-2B beads [66,67], having an apparent pore size distribution of 42, 31, and 75 nm, respectively. 

SEC has several advantages. The vesicular structure of exosomes is highly intact since they are not exposed to any extreme circumstances, such as centrifugal forces [68]. Because of its relatively inexpensive and quick separation, SEC is considered a feasible choice for bulk-scale exosome production.

### 2.5. Polymer-Based Precipitation Separation

This approach includes charge-based exosome precipitation using hydrophilic polymers like polyethylene glycol (PEG). PEG reduces exosome solubility by capturing water molecules and forcing exosomes to sink at a relatively low centrifugal force. Exosomes are precipitated when exosome-containing samples co-incubate with PEG solution (MW: 8000 Da). After incubating at 4 °C overnight, the deposited exosome can also be recovered or isolated by filtering or centrifugation. The following approach does not need advanced equipment and is reasonably simple to use with minimal downtime [69,70]. 

### 2.6. Magnetic Separation

Clayton et al. proposed a simple and quick approach for routine exosome separation and analysis based on immuno-magnetic isolation of exosomes containing human primary histocompatibility complex class II moieties [63]. This approach employs magnetic beads bounded with antibodies directed against proteins expressed on the exosomal surface. This method offers several benefits. Flow cytometry with fluorophore-conjugated antibodies can be utilized to investigate the bead-exosome complexes, allowing for fast quantitative analysis of the exosome surface make-up. On the other hand, these complex systems can be immunoblotted, and their composition is determined by treating them with an SDS sample buffer [62].

### 2.7. Acoustic Fluidic Separation

Acoustic fluidic separation is highly scalable, allowing for the manipulation of bioparticles ranging from nanoscale to microscale. Transducers built of piezoelectric materials are a practical approach to creating acoustic waves. When mechanical tension is applied, piezoelectric materials can generate electrical polarization, or mechanical deformation can be caused by electrical polarization [71]. Lee et al. used a standing surface acoustic wave separation instrument operating at 38.6 MHz to separate extracellular vesicles [72]. Exosomes were isolated from other extracellular vesicles using a cut-off size of 300 nm. Because of its biological acceptance also contactless separation advantages, the acoustic-fluidic separation technique has tremendous potential. The process output is limited to 0.40–0.43 L/min [72]. 

### 2.8. Dielectrophoretic Separation

Dielectrophoretic separation depends on the dielectric force acting on polarized particles in a non-uniform electric field. Nanoscale particles are attracted to the dielectrophoretic (DEP) high-field zones surrounding the circular microelectrode edge. The DEP low field zones amongst the electrodes attract cells and more prominent entities. Finally, the DEP field has little effect on tiny biomolecules, cations, and anions. A difference in dielectric properties between nanoparticles and the surrounding fluid (blood, plasma, serum, etc.) causes the DEP force [73]. The dielectric constant governs how rapidly charges travel via a medium when the external electric field changes. Transient dipoles form across the nanoparticles due to charges in the fluid medium and nanoparticles realigning themselves at various speeds in response to the shifting external electric field. These dipoles produce the forces that drag nanoparticles into the DEP high-field areas in a nonuniform electric field [74]. Although the requirement for electrothermal heating constraints this technique’s application, its advantages in speed and output might be considered.

### 2.9. Deterministic Lateral Displacement (DLD) Separation

Huang et al. pioneered DLD, a robust passive microfluidic particle separation approach that uses pillar arrays to separate particles depending on the dimensions [75]. DLD offers promise because of its low cost, robustness, and ability to precisely manipulate particles and efficiently separate them. DLD works at a low Reynolds number compared to other isolation methods and allows high dynamic size isolation, from millimeters to micro and nano sizes. It was recently shown that DLD could identify molecules according to morphologies, compressibility, and electrical characteristics. Because of separation’s excellent responsiveness, with a resolution limit of 20 nm. In microfluidic DLD, tilted pillar arrays are employed to create a fluid bifurcation and different streamlines between the gaps. The total fluid flux across each gap can be grouped by the periodicity (N), resulting in the conclusion that the DLD array’s periodicity equals the number of streamlines between each pillar. Fluidic forces and pillar barriers impact the particle fluxes in the DLD array. After a molecule enters the gap, any molecule with a small radius, when compared to the first streamline width, will follow the first streamline and travel in a zigzag pattern.

On the other hand, the molecule of size more significant than the 1st streamline moves straight and is forced against a pillar and displaced to the following streamline laterally [75]. Researchers encounter separation and clogging issues despite its ease of use and lack labels [76].

### 2.10. Microfluidic Devices

Using small samples and microfluidic devices, it is possible to automate the exosome separation and purification process [77]. These devices possess the unique feature of purifying EVs and their classes faster with high precision. Depending on their size, charge, surface properties, and interactions, these devices may combine sieving barriers, mixing chambers, and antibody-coated and functionalized layers to enable the separation of EV subclasses. These approaches are affinity-based [78,79]. Microfluidic devices are mainly used as diagnostic instruments because they specialize in small volumes (L). 

Example: It finds cell-specific EV enrichments associated with particular disease states.

Though it has excellent output in diagnostics, this method is suffering from significant limitations of non-scalability on large-scale diagnostics due to the absence of standardization of components and benchmarking [80]. 

The principles, advantages and disadvantages of exosome separation methods are given in Table 2.

## 3. Characterization of Exosomes

Exosomes are characterized based on their physical, chemical, functional, structural and biological [19]. Exosomes may need more characterization techniques for specific uses, for example, in enzyme replacement therapy (ERT), where they play the role of the vehicle to transport drug substances or biological compounds to the site of action. Exosomes must be created using precise procedures that consistently produce exosomes with composition, structural, and functional properties that fall within a specific range of acceptable ranges. The techniques used for the characterization of exosomes are described below.

### 3.1. Nanoparticle Tracking Analysis (NTA)

The nanoparticle tracking analysis (NTA) approach uses the particle light scattering and Brownian motion to quantify particle diameter. The hydrodynamic diameters are determined by following particles’ simultaneous, individual Brownian motion. Because each particle is photographed in distinct areas, it may be possible to discriminate between different sample sizes. NTA can also use fluorescent labeling to measure fluorescence and estimate the presence of antigens on exosomes [93,94]. 

### 3.2. Dynamic Light Scattering (DLS)

The physical technique of Dynamic Light Scattering (DLS), also known as “Photon Coherence Spectroscopy”, is used to determine particle size and dispersion. It works based on detecting particle light scattering and analyzing an optical signal to detect particles. This approach is suitable for predicting exosome size but can’t get source data. Moreover, the major shortcoming of this technique is for formulation containing particles of various sizes, significantly larger size particles that interfere with the detection of smaller particles. Hence, this technique’s obtaining size distribution is not perfect [95,96].

### 3.3. Atomic Force Microscopy (AFM)

Determining vesicle diameters using cryogenic transmission electron microscopy (cryo-TEM) is considered a gold standard technique, but problems are associated with it. The instrument’s cost, the knowledge necessary for sample preparation, imaging, and data interpretation, and a tiny number of particles consistently visible in images are all obstacles. Hence, the available and accessible substitute is atomic force microscopy (AFM) to overcome these challenges. AFM can provide valuable data on extracellular vesicles’ three-dimensional geometry, size, and other biophysical characteristics [97]. It also maps exosome mechanical properties and estimates their relative size distribution. It achieves sub-nanometer resolution imaging by scanning the surface with the tip of the cantilever beam. Structure, biomechanics, and abundance may be quantified and detected using AFM [98]. 

### 3.4. Microscopy Study

#### 3.4.1. Transmission Electron MICROSCOPY (TEM)

Transmission electron microscopy (TEM) examines the form and structure of particles using an accelerated electron beam. The images of exosomes obtained by TEM can estimate their size. Moreover, the morphology of exosomes can be impacted while preparing of TEM sample. To overcome this technique’s limitation, the advanced process “Cryo-TEM” was invented by scientists. Cryo-TEM is an advanced version that avoids sample preparation effects [19,99]. 

Spherical exomes and exosomes are different (heterogeneous) in shape, according to Colombo et al. [100]. However, the electron beam may be responsible for destroying biological samples in a few circumstances. The cup-shaped structure of isolated exosomes analyzed by TEM is the most prominent feature, and frozen exosomes are examined using cryo-TEM techniques to reveal round forms [101]. 

#### 3.4.2. Scanning Electron Microscopy (SEM)

Scanning Electron Microscopy (SEM) detects low-energy electrons ejected from only form proximity to the sample surface. It can give valuable sample data, including morphology, composition, surface texture, and roughness [102]. Thus, SEM provides information regarding exosome surface characteristics comprising size, shape and morphology. In a few cases, backscattered electrons (BSE) detector could also be helpful, especially for exosomes containing surfaces labeled with heavy metal [103]. It gathers electrons from more depths below the sample’s surface than scattered electrons and gives information about the composition and topography [104].

### 3.5. Enzyme-Linked Immunosorbent Assay (ELISA)

A plate-based enzyme-linked immunosorbent test identifies and measures proteins, peptides, hormones, and antibodies. It has been used to detect exosomes but needs samples in larger quantities and suffers from less responsiveness. With ELISA’s help, this assay is designed to accurately provide cancer exosome numbers [105]. It also determines exosomes from plasma, serum, and urine using different antibodies [106]. 

### 3.6. Fluorescence Correlation Microscopy (FCM)

Exosomes are captured using a particular antibody, tagged with a fluorescent dye and measured by a plate reader in microfluidic-dependent Fluorescence Correlation Microscopy (FCM). A microfluidic chip used for immunocapture and quantitative exosome analysis can be developed using FCM [107].

### 3.7. Colorimetric Detection

A chromogenic substance’s color intensity determines the particles in calorimetric detection. Exosomes were captured using microfluidic technologies, and exomes were detected and quantified using ELISA. Scientists and researchers have successfully utilized this technology to detect exosomes from cancer cells [108,109,110].

### 3.8. Surface Plasmon Resonance (SPR) Detection

Scientists and researchers have successfully utilized this technology to detect exosomes from cancer cells. Exosomes are studied using a nano-plasmonic exosome (nPLEX) created by modifying a nano substrate to improve detection performance. We will be able to functionalize every nanopore depending on the nPLEX chip. Hence, the distinct nano-size pore can bind specific exosomes. It uses a microfluidic-based SPR device to detect distinct exosomes and assess several exosomal proteins [111,112]. 

### 3.9. Nuclear Magnetic Resonance (NMR) Detection

Nuclear Magnetic Resonance (NMR) is mainly utilized to analyze chemicals. To evaluate the quantity and presence of proteins in exosomes, micro-NMR techniques have also been developed. Systems can detect exosomes after concentrating microvesicles containing immunogenic nanoparticles via filtering. The number of exosomes is quantified using a signal-to-noise ratio [113,114]. 

All the characterization techniques are summarized in Table 3.

## 4. Exosomes as Drug Delivery Vehicle

Many advanced drug delivery systems like polymeric nanoparticles and liposomes encapsulate drug substances. They are most frequently used to deliver anticancer, antiviral, and antifungal drugs. Still, the significant concerns are their compatibility with living tissue, extended stability, and the potential to attack the host’s immune system for extended periods [115]. A recent advancement was made in the drug delivery science is exosomes; it is a novel nanoscale delivery system with many advantages, such as biocompatibility, biodegradability, less toxic, specificity to the target cells, small size, promotes plasma membrane fusion, among different cells, longer half-life, low-uptake machinery, capability to pass contents from one cell to another cell, and do not produce an immune reaction and the unique feature that they have more tendency to accumulate in the cancerous cell than normal cells make it a good choice. By understanding this, we can modify the exosomes, which help in the delivery of protein, peptide, DNA, RNA, small molecules, large molecules, nucleic acid, etc. [116].

### 4.1. Small Molecules

Exosomes are also attractive small-molecule drug delivery carriers because of their small size, lower toxicity, and biocompatibility than other nanocarriers wiz, liposomes, niosomes, etc. Exosomes also act as nanoparticles that can carry their cargo to receptor cells and also helps in cellular exchange that can be used as a carrier for drug delivery. Exosome-encapsulated APIs show superior PKPD features and in vivo anti-neoplastic activity compared to free drugs. Exosome-loaded small compounds have been reported to offer superior therapeutic advantages in a few investigations; for example, exosomes containing doxorubicin were developed and tested for distribution along with fast cellular absorption to the intracellular fluid [117]. Compared to free doxorubicin and its liposomal forms, exosomes of the doxorubicin demonstrated better in vitro potency in various cell types and increased cell absorption and biodistribution. Several tests were then conducted to demonstrate that curcumin incorporation within the exosome enhances solubility, stability, and bioavailability [118]. 

Exosomal curcumin’s anti-inflammatory efficacy was also tested in vitro and in vivo. *In vitro*, exosome containing curcumin inhibited the release of inflammatory cytokines such as interleukin-6 (IL-6) and tumor necrosis factor (TNF-), implying that exosome containing curcumin has anti-inflammatory characteristics. Animal model of lipopolysaccharides the septic shock created in vivo, and mice treated with exosomal curcumin outlived those treated with just curcumin. Finally, exosomal curcumin was found to lower the number of CD11bGr-1 cells in the lungs, which improves the response to lipopolysaccharide-induced septic shock and causes acute lung inflammation. The following work demonstrated that exosomes might transport extremely hydrophobic medicines like curcumin, boosting their anti-inflammatory properties [119]. Exosomes transport small molecular medications transverse to the blood-brain barrier (BBB) and increase therapeutic characteristics. Indeed, 98 percent of powerful centrally-acting drugs cannot pass through BBB, and their in-vitro efficacy has not been replicated in clinical trials. Most of the nano-formulations have been used to tackle difficulties related to medication permeability across the BBB. Nanotoxicity and fast drug elimination by the mononuclear phagocyte system (MPS) were additional problems. Polyethylene glycol (PEG) reduces MPS medication absorption to compensate for these issues. This, however, resulted in less contact between target cells, resulting in decreased drug distribution throughout the brain. In this scenario, exosomes, naturally produced by the body’s cells substance, may be made to pass the BBB, increasing drug delivery to the brain while lowering MPS drug clearance [81].

### 4.2. Large Molecule (Protein and Peptide Delivery)

Large molecules, such as proteins and peptides, are delivered using exosomal drug delivery systems. Exosomes were researched to transmit biological materials for diverse tasks such as therapeutic and diagnostic reasons, as they were found to remove the protein, lipids, and nucleic acid unwanted for cells [120]. Since research has shown that exosomes separated from most cells are intrinsic carriers for endogenous protein molecules, exosomes are currently portrayed as ideal carriers for protein and peptide molecules [119]. Recent research, for example, in diseases like Parkinson’s, have shown that exosomes containing the antioxidant protein catalase were effectively carried across the BBB, resulting in a better disease state [115].

### 4.3. Nucleic Acids

Exosomes have also been shown to transport nucleic acids like DNA, RNA, mRNA, miRNA etc., to specific cells in the body, producing genetic alterations in both normal and pathological processes. They target cytoplasm and alter cell function that can be used for the treatment of various diseases. Exosomes piqued researchers’ interest in gene therapy treatment alternatives because of their natural carrying capacity of genetic material. Exosomes have been used in experiments to administer therapeutic genetic substances that influence gene expression in specific illnesses and promote genetic treatment [120]. It also can carry different biological components such as lipids, proteins and nucleic acids. It contributes to the intercellular communication process [121,122,123,124,125]. 

### 4.4. Small Interfering RNAs (siRNAs)

SiRNA is a kind of RNA that disrupts genes of interest in genetic treatments. However, it has limited stability and degrades systemic circulation. On the other hand, storing and giving siRNA to specific cells can act as a therapeutic carrier and helps to overcome this barrier. Most research has been done to evaluate effective siRNA carriers to target cells [126]. Exosomes were employed to distribute siRNA because they show no immune response and naturally transport RNA from cell to cell. Researchers discovered that endothelial exosomes could deliver siRNA to endothelial cells. Some experiments used exosomes from dendritic cells to electroporate loaded exogenous siRNA to achieve tissue-specific targeting.

In contrast, dendritic cells were modified to produce exosomal surface component Lamp2b. Several research in recent years has revealed the effective transport of siRNA to target cells. It has also been discovered that the loaded siRNA is delivered to cancer cells through exosomes. Exosomes obtained by centrifugation by HEK-293 cells were tagged with fluorescent dye before being electroporated into exosomes. After loading it into the exosomes, gel filtering was used to remove the excess siRNA. The findings showed that siRNA encapsulation was adequate, with a high exosome production and successful transport into cancer cells [113].

### 4.5. MicroRNA (miRNA)

miRNA is a short non-coding RNA in eukaryotic cells and contains non-proteinaceous nitrogenous bases. miRNAs regulate post-translational gene expression by binding to complementary sites on targeted mRNA. Because exosomes naturally transport miRNA, they can be used to therapeutically transfer miRNA to selected cells [127]. The research used exosomes to deliver miRNA to breast cancer cells targeting the epidermal growth factor receptor (EGFR). EGFR expression was high in various human cancers derived from epithelial cells, indicating that EGFR ligands might be used as cancer treatment targets. Exosomes were created to deliver 5-fluorouracil (5-FU) with microRNA-21 inhibitor oligonucleotide (miR-21i) to HER2, expressing cancerous cells resulting in cell cycle arrest and decreased tumor growth and increased apoptosis. They restored PTEN and hMSH2 levels, which are miR-21 regulatory targets. In 5-FU tolerant colon cancerous cells, combining miR-21i with 5-FU delivery in exosomes successfully overcame drug resistance and efficiently boosted cytotoxic activity [128]. Exosomes generated from MDA-MB-231 cells were employed in another investigation to treat NSCLC (Non-Small-Cell Lung Cancer). Because of a link between the cancer cell surface protein C (SPC) and the overexpressed integrin 4 (found on exosomes), NSCLC absorbed exosomes (231-Exo) preferentially. The antisense oligonucleotide of miRNA-155 was incorporated into exosomes by RBCs with the help of an electroporator, which resulted in enhanced therapeutic activity on lung diseases. Exosomes (miR-126:231-Exo) were loaded with miRNA-126, which decreased cell proliferation and migration by interfering with the PTEN/PI3K/AKT signaling pathway. An in vivo investigation revealed that it inhibited lung cancer metastasis in mice [126].

### 4.6. Clustered Regularly Interspaced Short Palindromic Repeats (CRISPR)/CRISPR Associated Protein 9 (Cas9) System

Scientists recently employed CRISPR/Cas9 systems to cure numerous hereditary disorders like cancer by repairing, eliminating, or suppressing disease-related genomic abnormalities. It is a two-part system for the deletion, insertion and modification of particular genes. It is comprised of the Cas9 protein and a single-guide RNA (sgRNA) (RNA-guided endonuclease) [22]. To be effective in gene editing, the CRISPR/Cas9 system must be supplied to receptor cells in a certain way. The researchers employed a Liposome-exosome hybrid nano system to introduce the CRISPR/Cas9 system within MSCs. Ultracentrifugation was used to separate exosomes from HEK293FT cells. Exosomes were incubated with liposomes to form hybrid exosomes; before being delivered to MSCs, significant DNAs were enclosed within exosomes. They were capable of transporting hybrid nano-systems into MSCs and controlling the expression of targeted genes [129]. Exosomes have been discovered to be ideal carriers for cancer treatment due to cell tropism-induced selective accumulation. Exosomes containing CRISPR/Cas9 reduced the formation of poly (ADP-ribose) polymerase-1 (PARP-1), causing ovarian cancer cells to die. CRISPR/Cas9 genetic material transformation improved cisplatin chemosensitivity, resulting in synergistic cytotoxicity [127]. 

**Figure 3:**
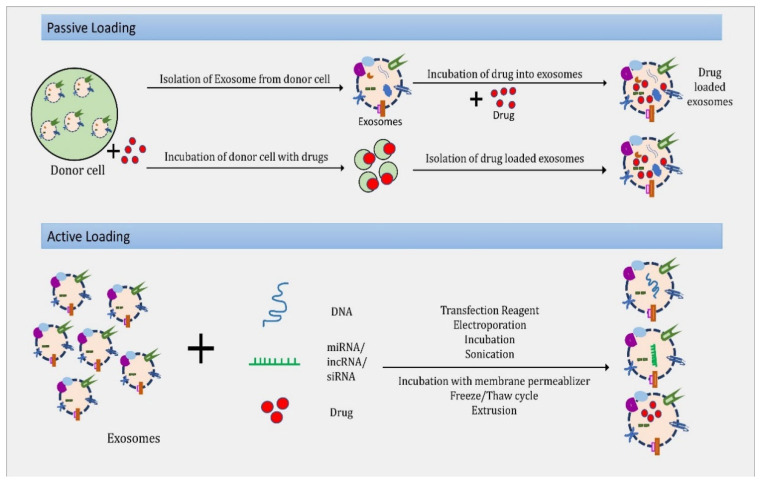
Passive and active loading methods of exosomes.

The drug-loading methods (passive and active) of exosomes are shown in Figure 3.

## 5. Therapeutic Applications of Exosomes

### 5.1. In Cancer

The potential of exosomes for use in cancer therapy has attracted much interest in recent years. Because of their biological signals, exosomes can be seen as an enhanced drug delivery method for cancer treatment. Compared to synthetic drug carriers like synthetic polymer-based nanoparticles, micellar systems, liposomes, etc., exosomes have unique, innate properties, including tumor targeting and interaction with tumor vessels and stroma, which makes them capable of delivering drugs deep inside tumors despite tissue and cellular barriers [130]. Exosomes can be indicated as a less drastic approach to diagnosing cancer. In blood, urine, plasma, and other samples, various proteins (ZIP4, HSP60, EphrinA2 etc.) are exosome biomarkers for different cancer types [131].

Kamerkar et al. presented an intriguing study using modified exosomes (exosomes) for cancer prevention and treatment [132]. They discovered that exosomes had a longer retention time in the circulation of mice than liposomes, which they attributed to exosomes’ defense against phagocytosis by monocytes and macrophages via CD47. Investigators additionally discovered that engineered exosomes containing small interfering RNA specialized for malignant KrasG12D, a typical pancreatic cancer mutation, were more successful in targeting oncogenic KRAS than liposomes.

Furthermore, the administration of modified exosomes (siKrasG12D iExo) significantly reduced tumors and improved continued existence in a mouse model. Still, CD 47 destruction-engineered Exosomes (CD47 k/o siKrasG12D iExo) failed to inhibit tumor growth and enhance life expectancy. MSCs derived from adipose tissue and injected with a miR-122 interpretation plasmid can produce miR-122-containing exosomes that can be applied to transport miR-122 to liver cell cancer and improve its treatment responsiveness [133]. The CRISPER/Cas9 plasmid can use cancer cells’ exosomes as a transporter, allowing it to transfect ovarian cancer cells successfully. As a result, Poly (ADP-ribose) polymerase-1 activity has been lowered, and cell death in overproliferated tumor cells also increased. 

In addition, it boosted cancer cells’ susceptibility to chemical treatments [134]. Cancer’s metastasis, in addition to cancer itself, is a significant hazard to human health. Exosomes are well-known as critical mediators for cell–cell communication, but they may also contribute to cancer spread in the presence of malignancy. Exosomes produced from organotypic tumor cells merged preferentially with indigenous cells, according to Hoshino et al., partly due to exosome integrins [135]. According to the researchers, A6b1 and A6b4 exosomal integrins have been associated with pulmonary tumor growth, whereas integrin avb5 was linked to liver tumor growth. As a result, inhibiting the integrins of exosomes may be an approach to preventing tumor metastasis. Exosomes, released by extremely invasive bone cancer cells, contain TGFb type that promotes the paracrine function related to MSC’s IL6 and contributes to tumor formation and metastasis [136]. Exosomes released by cancer cells, enclosed in a large proportion of VEGF and IL-8, add a significant role in cancer neurovascular genesis and progression [137]. As a result, limiting exosome formation, release, and endocytosis can help to prevent tumor spread. Dynamin is required for EV production and is closely linked to exosome endocytosis in breast cancer cells [138,139]. Dynasone, a dynamin inhibitor, can indirectly limit the quantity of exosome absorption by cancer cells by inhibiting exosome formation [140]. Exosome activity and endocytosis can both be reduced by dynamin siRNA transfection [141].

### 5.2. In neurological Diseases

#### 5.2.1. Parkinson’s Disease

After Alzheimer’s (AD), Parkinson’s disease (PD) is the second most common neurological condition. Protein aggregates, which are primarily composed of misfolded and fibrillary forms of α-synuclein (α-syn), and are found in neuronal soma and axons, are also present. It is distinguished by bradykinesia, resting tremor, muscular tone stiffness, and cognitive engagement. These symptoms, along with the distribution pattern of α-syn aggregates, are a characteristic of Parkinson’s disease pathogenesis [142]. Exosomes carrying α-syn generated by damaged neurons might be transported from neuron to neuron, causing α-syn to spread and form neuron to glia, causing an immune reaction to be activated. Exosomes carrying inflammatory mediators generated by activated glial cells can then be transported from glia to glia, propagating the inflammatory response. As a result, the spread of inflammatory mediators and enhanced neuroinflammation may lead to neuronal dysfunction and disease progression. The research suggests that exosomes play varying roles in Parkinson’s disease, owing to unknown features of exosome complexes [143].

Furthermore, exosomes may be used for diagnostic or novel treatment applications in Parkinson’s disease research. Identifying novel biomarkers to monitor the course of neurodegenerative illnesses might shorten clinical trials, lowering the costs and time required to bring vital medicines to patients. Exosomes may offer therapeutic promise in treating neurodegenerative diseases; currently, there are no medicines that can prevent Parkinson’s disease because the BBB impedes most pharmaceuticals, necessitating the development of novel drug delivery techniques [144]. Exosomes have been proposed as a therapeutic delivery mechanism due to their homing ability; they can convey their cargo to particular destinations across extended distances. However, various issues must be addressed before exosomes may be presented as biomarkers or a medication delivery strategy for PD in the clinical context [145].

#### 5.2.2. Alzheimer’s Disease (AD)

Alzheimer’s disease (AD) is a degenerative neurological disease marked by 50–80% of cases of dementia due to memory and other cognitive deterioration. Alzheimer’s is a slowly progressing brain illness, with pathological alterations occurring many years before clinical symptoms. However, besides medications that only temporarily reduce symptoms, a better knowledge of risk factors and molecular processes underlying AD pathogenesis can lead to new ways to prevent and treat the disease [146]. Exosomes centrally use the cerebrospinal fluid to transport signals between cells and throughout the brain (CSF). As a result, exosomes and their contents are thought to function in nervous system physiology and neurodegenerations like Alzheimer’s disease. Exosomes produced from different cells of CNS and their roles are as follows:

Exosomes produced by astrocytes has connected to axon development, neurogenesis, synaptic transmission, neuroinflammation, and neuronal survival.

Exosomes generated by oligodendroglia may help develop neurite, stress resistance and neuronal function.

Exosomes from microglia contain various proteins, chaperones, tetraspanins, surface receptors, and miRNAs linked to neuronal survival, neurite outgrowth, and neuro-inflammatory reactions [147]. Using exosomes as an indicative biomarker for Alzheimer’s is gaining popularity [148].

#### 5.2.3. Epilepsy

Exosomes are intercellular messengers that deliver a molecular payload that can change the phenotypic and gene expression of the recipient cell. Researchers examined changes in exosomes carrying microRNAs formed by tissue that causes seizures in the tuberous sclerosis complex (TSC), a various hereditary condition defined by tuber lesions in the brain. Exosomes were isolated from epileptogenic and non-epileptogenic TSC tubers, and a short RNA sequence was used to uncover differences in microRNA cargo [149]. They discovered various microRNAs (miR-142-3p, miR-223-3p, and miR-21-5p), which considerably present higher in epileptogenic tubers and comprise genetic patterns, resulting in the activation of Toll-like signals (TLR7/8), triggering an inflammatory neuronal pathway [150]. In cultured cells, exosomes from epileptogenic tubers triggered crucial pathways such as innate immunological signaling, immune system activation, and essential nodes like SQ STM1 (p62) and CDKN1A (p21) for signaling. In epileptogenic tissue, the genetic material activated in vitro was highly elevated. These findings provide an additional understanding of the role of exosomes and non-coding RNA cargo in the epileptic neuroinflammatory cascade, potentially leading to the development of new biomarkers and treatments for drug-resistant epilepsy [151].

#### 5.2.4. Huntington’s Disease

The HTT gene’s CAG mutation results in Huntington’s disease (HD), leading to a mutant huntingtin protein with a polyglutamine expansion at the N-terminus (mHTT). The modified protein aggregation, linked to cortical atrophy and a specific loss of medium spiny neurons in the striatum, is linked to cognitive impairments and involuntary movements. Exosomes are hypothesized to play a role in the protein and RNA levels of mHTT protein proliferation within cells. Researchers observed that introducing exosomes from HD patients’ fibroblasts into the ventricles of a newborn mouse brain triggered HD-like behavior and pathology. This result, however, requires additional validation since other components may co-isolate with exosomes [152].

On the other hand, exosomes produced by astrocytes and stem cells generated from adipose tissue have been shown to exhibit neuroprotective properties [152]. HD 140Q knock-in (KI) mice striatum was injected with astrocytic exosomes, reducing mHTT aggregates’ density. Interestingly, no mHTT protein was found in exosomes generated via primary astrocytes by 140Q KI mice, implying that astrocyte-derived exosomes could be employed to treat HD. In an in vitro HD model, exosomes produced by adipose-derived stem cells are considered to generate neurotrophic factors, reduce mHTT aggregates, and kill cells. Exosomes effectively deliver oligonucleotide therapies in HD (miRNA and siRNA) [153].

#### 5.2.5. Stroke

Identifying appropriate molecular markers for stroke detection and therapy assessment is one of the most pressing challenges. The central nervous system produces and secrets exosomes, which can be found in blood or CSF after crossing the BBB. In response to a stroke, blood cells and endothelial cells release exosomes into circulation. They help improve the prolonged protection of neurons after a stroke by encouraging neuron repair [154]. In addition, they promote angiogenesis, neurogenesis, and axon dendrite remodeling. Exosomes released by brain endothelial cells can help brain reconstruction during stroke recovery by interacting with the central nervous system [155].

#### 5.2.6. Amyotrophic Lateral Sclerosis

ALS is a disease that involves proteins, such as superoxide dismutase-1 (SOD1) and TDP-43, causing neurodegeneration in the cortex, brainstem, and spinal motor neurons. The condition is characterized by protein malfunction missing the nucleic acids [156]. They inhibited exosome synthesis by silencing GW4869 or RAB27A genes, which caused TDP-43 aggregation in TDP-43A315T transgenic mice Neuro2a cells. Exosomes are primary inflammatory mediators of blood mononuclear cells. This shows that exosomes linked to ALS exist in the peripheral circulation and interact with other cells. In addition to the TDP-43 protein, other compounds found in related exosomes could be used as a biomarker for ALS [157].

### 5.3. Inflammatory Disease

Inflammation is an immunological response that protects the host against infections or injury. However, uncontrolled inflammation can lead to a variety of disorders. Innate immune factors such as cytokines, chemokines, and innate immune cells are the primary mediators of inflammation. Exosomes are gaining traction as a cutting-edge therapeutic method for treating an overactive immune system. Exosomes are secreted by most cells and can have powerful immunomodulatory functions depending on the kind of cell. Exosomes play a role in inflammatory processes that are significant in diseases like carcinoma, IBD, diabetes (type 2), obesity, rheumatism, and neurological disorders [158]. The discovery of a link between inflammation and changes in some exosomal cargos’ type or expression level is the first step toward finding potential new biomarkers of inflammatory disorders. The SLC22A5 transport protein, whose exosome expression is controlled by the INF-α, is a unique and intriguing exosome cargo. As a bio-marker carrier, exosomes are easy to collect from bodily fluids like salivary secretion and urine, and they may give a non-invasive technique of screening for diseases. According to a stream of research, exosomes play a significant role in shaping the inflammatory surroundings of tumors.

Exosomes secreted by patients with liver metastases contained significantly more Migration Inhibitory Factor (MIF) than patients without metastatic disease. As a result, exosomal MIF could be used as a predictor of PDAC hepatic metastasis advancement. IBDs are chronic diseases caused by a failure to maintain intestinal homeostasis in genetically predisposed people. Many strategies have been proposed to support immuno-tolerance via controlling activated T-cells. One includes exosomes’ immunosuppressive action [159]. Transferring normal intestinal exosomes into IBD mice reduces the extent of the disease. Exosomes from IBD patients, on the other hand, stimulate an increase in the pro-inflammatory IL-8 when infused into the human colonocyte cell line DLD-1. Macrophages are also necessary for intestinal homeostasis, and their activity has been linked to IBD pathogenesis [159].

### 5.4. Autoimmune Disease

Exosomes contain various bioactive compounds such as inflammatory mediators, allergenic molecules, cellular signaling factors, HSPs, different ribonucleic acids, or molecules with functionalities such as binding agents, costimulatory molecules, linkers, receptors, and so on. Exosomes can deliver functional microRNAs and circular RNAs to a specific site, resulting in immune activation and inhibition [160].

#### 5.4.1. Exosome’s Role in Rheumatoid Arthritis and Joint Diseases

In rheumatoid arthritis (RA) and other joint illnesses, exosomes released by invading inflammatory cells are generally harmful. In an inflammatory environment, synoviocytes secrete exosomes, which cause chondrocytes to release extra allergy mediators and metabolic proteins, leading to cartilage deterioration. Furthermore, exosomes found in RA patients’ synovium contain citrullinated autoantigens, cause inflammation, and are most likely produced by proliferating synoviocytes. Exosomes from infiltrating neutrophils into inflammatory joints, on the other hand, protect chondrocytes via a TGF-1-mediated mechanism. Exosomes carrying regulatory FasL and TRAIL generated by activated T-cells in the synovium may help prevent autoimmune damage in rheumatoid arthritis [161].

#### 5.4.2. Exosome’s Role in other Autoimmune and Chronic Inflammatory Diseases

In multiple sclerosis, exosomes have been found to cross the blood–brain barrier and aid in the transport of brain antigens to the periphery by immune system antigen-presenting cells (MS). On the other hand, exosomes produced biologically in the CNS appear to have a favorable effect on tissue equilibrium, promoting regeneration and neuroprotection [162].

Chronic inflammatory lung disease is another illness to which exosomes have been linked. In the case of chronic obstructive pulmonary disease (COPD), caused mainly by smoking, exosomes from lung cells consist of miR-210, which helps reduce Atg7 expression, blocks self-eating, and induces myofibroblast growth and scarring of tissue [160].

### 5.5. Renal Diseases

The kidney is one of the most critical organs in the human body, as it is responsible for maintaining and controlling homeostasis. Exosomes have been postulated to perform functions in cell–cell contacts within nephron segments, influencing renal physiology [163]. The role of exosomes in renal functioning has been studied in vitro and in vivo. Exosomes generated from various parts of the neuron have been depicted in several studies. Aside from their role in renal physiology, urinary exosomes also serve as immunological effectors, protecting the urinary tract against bacterial infection. Multiple innate immune proteins, such as lysozyme-C, dermcidin, mucin-1, calprotectin, and myeloperoxidase, are found in intact human urine exosomes, which block the growth of infective and non-infective strains of *E. coli* [164]. Table 4 shows the role of exosomes in renal diseases.

### 5.6. Cardiovascular Diseases

**Figure 4:**
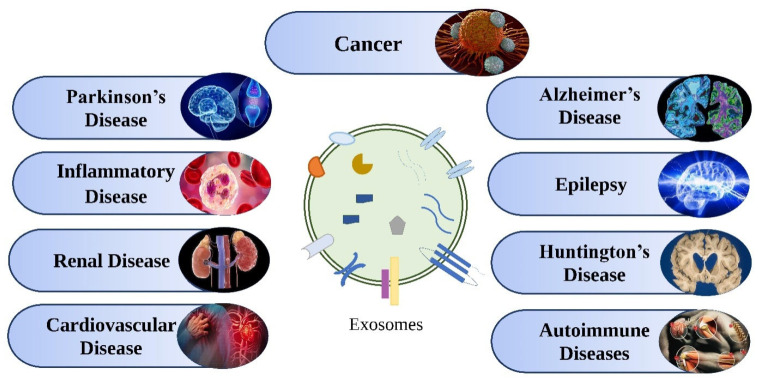
Therapeutic applications of exosomes in various diseases.

In the context of cardiovascular diseases, growing evidence suggests that these nanovesicles play a role in cardioprotection and, thus, their possible future use as a therapeutic tool for various cardiovascular disorders such as coronary artery disease, coronary heart failure, cerebrovascular disease, and other cardiac diseases. Exosomes have been shown to mediate communication in cardiac tissue between different types of cells [165]. HIF-1, for example, may be present in cardiac exosomes in less oxygen; this might increase Hsp2049 synthesis and start the formation of new blood vessels by boosting the expression of VEGF receptor-2 in endothelial cells. Exosomes produced from stem cells have been shown in studies to have a significant role in heart healing. The role and potential of exosomes in disease treatment and drug delivery is as shown in Figure 4.

Exosome examples in various diseases as advanced drug delivery carriers as shown in Table 5.

## 6. Challenges Associated with Exosome-Based Drug Delivery

Though exosomes have many benefits as drug delivery vehicles, several challenges must be overcome. The first main limitation of using exosomes as a pharmaceutical is its difficulty in extraction and separation methods causing low extraction yield [179]. If exosomes are exploited as drug delivery carriers on a commercial scale, high-quality exosomes must be isolated and separated in sufficient quantities. There is currently no perfect purification method for isolating and separating high-purity exosomes. The efficiency of available separation and isolation methods resulted in low yield and are costly for scale-up manufacturing.

Another disadvantage of exosomes is their limited encapsulation and loading potential. Exogenous hydrophilic macromolecules must be released, and undesirable cargo chemicals in exosomes must be resolved [180]. In addition, exosomes have a limited capacity for loading exogenous substances. This could be because exosomes are part of the parent cell from which they are formed [141]. Exosome biology is well understood, yet due to its diverse character as a result of biomolecules, exosomes can cause immunogenic reactions [127].

Despite having a distinct lipid and protein composition, exosomes are rapidly removed from systemic circulation after in vivo delivery [181]. Fewer than 5% of the injected exosomes remain in the bloodstream three hours after injection, just like phosphatidylcholine/cholesterol liposomes. Capturing exosomes by macrophages accounts for the quicker clearance of exosomes in vivo [97,182]. When injected intravenously, exosomes produced by B16BL6 cells were promptly removed from the body by splenic and hepatic macrophages [183].

Exosomes are rarely studied as a promising carrier for drug delivery in “non-conventional” clinical uses such as conjunctival, ophthalmic, transmucosal, and other areas. Understanding how exosomes can be used in drug delivery systems presents many challenges such as permeation across physical hurdles like tight epithelial junctions in various tissues and evading removal by local tissue fluid and enzymes under diseased conditions.

Unlike polymeric nanoparticles or liposomes, exosomes can circumvent the endosomal and lysosomal routes and transfer payload straight into the cytoplasm. Furthermore, a lack of understanding of exosome nature and role in general illness and health situations makes long-term safety and therapeutic success challenging to anticipate. Various obstacles exist to understanding exosomes in active loading and drug delivery assembly.

Yet another challenge arises due to the heterogeneity of exosomes due to their varied size, composition, function and cellular origin, which adds complexity to their characterization. Accurate and precise quantitative analysis of exosomes needs to be established, especially in terms of their purity, morphology, loading effectiveness and functional characteristics. Also, a significant effort must be made to address both technological and regulatory issues to speed up the effective translation of exosome-based therapies for the benefit of millions of impacted people [80].

## 7. Future Perspectives

Exosomes are currently considered to be potential drug delivery systems in the current scenario, providing new avenues to handle different diseases and disorders. However, our existing knowledge of exosomes may be insufficient and incorrect, making the development of exosomes for varied purposes. It is critical to consider exosome manufacturing, which should be plentiful enough for further study or therapeutic uses. Exosome quality and purity standards should be tightened to enhance their therapeutic benefits. Although assessment methodologies have helped identify exosomes from other fractions, exosome-specific assays still need to be developed and validated. To manage their tactfulness and composition, exosome storage conditions (temperature or pH) are still challenging to determine. Adding drugs would complicate keeping the stability of exosome formulations even further. Only a few research findings suggest that exosomes eventually lead to FDA-approved nanomedicines. Exosomal formulations must adhere to GMP regulations, which include protocols for quality control, standard cell culture techniques, downstream exosome isolation, and source selection for exosomes. Thus, new approaches and principles are required to address these pitfalls, which could increase the success of exosome-to-medicine translation. 

## Figures and Tables

**Table 2:** Principles, advantages and disadvantages of exosome separation methods [80,81].

Method	Principle	Advantages	Disadvantages	Reference
Ultrafiltration	Separation is based on molecular weight and size.	It is faster, requires no special equipment, and is easier to handle than ultracentrifugation.	Exosome clogging and entrapment in filters lead to a poor recovery rate, deformation, and damage to large EVs due to the force required to drive through the filters.	[82,83]
Immunological Separation	Exosomes are captured via an antigen-antibody response.	The method saves time by separating bodily fluids immediately, isolation of high purity, and a simple process	Exosome separation on a large scale is impossible due to the high reagent cost, poor capacity, and yield. Non-physiological salt and pH conditions are required.	[84,85]
Ultracentrifugation	Sedimentation coefficient of exosomes and other substances in sample	Exosomes may be produced in enormous quantities, with great separation purity.	Time required (>4 h), low recovery rate (5–25%), and poor reproducibility make it unsuitable for clinical diagnosis.	[82,86]
Size-Exclusion Chromatography	Utilizes a column of porous polymeric beads to separate exosomes based on size.	High yield and purity	Expensive, time-consuming post-isolation analysis and column contamination.	[87,88]
Polymer-based precipitation separation	Hydrophobicity	Simple procedure with a small sample volume	Long run periods and post-separation cleaning are required.Non-exosome contaminants are co-precipitated in the sample.	[89,90,91]
Magnetic separation	Magnetic Force	The contactless separation, high specificity, and high throughput	Magnetic labeling	[66,67]
Acoustic fluid separation	Separation is based on size	A technique that is label-free, contactless, and quick.	Separation is not extensively used yet.	[88,92]
Dielectrophoretic separation	Polarized particle’s size and electric properties	Characteristics of label-free, contactless, fast, and high-throughput	Low resolution, low purity, Joule and electrothermal heating problems	[77,78]
Deterministic Lateral Displacement Separation	Critical size for particle separation	Label-free, easy to use	Low separation purity, clogging	[79]
Microfluidic devices	Separation based on size, charge, surface properties and interactions	Fast, High precision	Non-scalability on large-scale diagnostics	[80]

**Table 3:** Characterization techniques of exosomes.

Sr. No.	Characterization Technique	Principle	Application	References
1.	Nanoparticle Tracking Analysis (NTA)	Particles’ light scattering and Brownian motion	Quantify particle diameter Estimate the presence of antigens on exosomes.	[93,94]
2.	Dynamic Light Scattering (DLS)	Particle light scattering and optical signal	Determine particle size and dispersion.	[95,96]
3.	Atomic Force Microscopy (AFM)	Surface sensing, detection, and imaging	3D geometry, size, and other biophysical characteristics. Mechanical properties.	[97,98]
4.	Microscopy study Transmission Electron Microscopy (TEM) Scanning Electron Microscopy (SEM)	Accelerated electron beam Low-energy electrons are ejected from only form proximity to the sample surface.	3D form, size and structure of particles. Surface characteristics comprising size, shape and morphology.	[19,101,102]
5.	Enzyme-linked Immunosorbent Assay (ELISA)	Plate-based enzyme-linked immunosorbent test	Identifies and measures proteins, peptides, hormones, and antibodies; also used to determine exosomes from plasma, serum, and urine using different precise antibodies.	[105,106]
6.	Fluorescence Correlation Microscopy (FCM)	Antibody tagged with a fluorescent dye and measured by a plate reader in microfluidic-dependent FCM.	Immunocapture and quantitative analysis	[107]
7.	Colorimetric detection	Determines the particles in calorimetric detection, quantified using ELISA.	Utilized to detect exosomes from cancer cells.	[26,109,110]
8.	Surface Plasmon Resonance (SPR) detection	Microfluidic-based SPR device.	Improve detection performance by nano-plasmonic exosome (nPLEX) created by modifying a nanosubstrate. Able to functionalize every nanopore depending on the nPLEX chip.	[111,112]
9.	Nuclear Magnetic Resonance (NMR) detection	Micro-NMR technique	Assess the number and presence of proteins in exosomes. Detect exosomes after concentrating microvesicles containing immunogenic nanoparticles via filtering.	[113,114]

**Table 4:** Role of exosomes in renal diseases [164].

Kidney Disease/Disorder	Exosome’s Role	Exosome’s Source	Main Findings
Renal cell carcinoma (RCC)	Therapeutics	RCC cell line Pathogenic	CD8 + T-cells activated by exosomes generated from RCC cells combined with GM-CSF and IL-12 showed autologous anti-cancer activity.
	Biomarker	Urine (human)	Urinary exosomal miR-126-3p in combination with miR-449a or miR-34b-5p might distinguish ccRCC from healthy people. Urinary exosomal miR-126-3p in combination with miR-486-5p in urine might distinguish ccRCC from benign tumors.
Kidney stone disease	Pathogenic	Urine (human)	Urinary exosomes were produced in larger quantities by stone formers.
Renal fibrosis	Therapeutics	MSCs (human)	Exosome miR-let7c generated from MSCs has reduced fibrosis in renal tubular epithelial cells.
Polycystic kidney disease	Pathogenic	Urine (human)	Multiple PKD-related gene products were excreted into the urine via exosomal secretion.

**Table 5:** Use of exosomes as drug carriers in various diseases adopted with kind permission from reference [35].

Drug	Type of Drug	Disease Model	Therapeutic Effect	Exosomes Origin	Drug Loading Method	Reference
**Paclitaxel**	Small molecule drug	Autologous prostate cancer	Enhanced drug cytotoxicity to cancer cells	Prostate cancer cell lines (PC-3 and LNCaP)	Co-incubation	[164]
SiRNA	Genetic substances	Alzheimer’s disease	Specific gene knockdown after specific siRNA delivery to the brain	Dendritic cells (gene engineered to express Lamp2b)	Electroporation	[166]
Paclitaxel	Small molecule drug	Pancreatic adenocarcinoma	Inhibited growth of human pancreatic adenocarcinoma cell	Mesenchymal stromal cells	Co-incubation	[167]
Curcumin	Small molecule drug	Lipopolysaccharide-induced shock	Enhanced anti-inflammatory activity	Mouse lymphoma cell (EL-4) and RAW 264.7 cells	Direct mixing	[168]
Doxorubicin	Small molecule drug	Breast cancer	Specific drug delivery to the tumor site and inhibited tumor growth	Immature mouse dendritic cells transfected with the vector expressing iRGD-Lamp2b fusion proteins	Electroporation	[169]
Paclitaxel	Small molecule drug	Cancer with multiple drug resistance (MDR)	Overcome MDR cancer in vitro and in vivo	RAW 264.7 cells	Sonication	[170]
Paclitaxel	Small molecule drug	Pulmonary metastases	Reduced pulmonary metastases in vitro and in vivo	RAW 264.7 cells	Sonication	[171]
Curcumin	Small molecule drug	Brain tumor and autoimmune encephalitis	Inhibited brain inflammation and delayed brain tumor growth	Tumor cells (GL26-Luc, BV2, 3T3L1, 4T1, CT26, A20 and EL-4)	Direct mixing	[172]
Dopamine	Small molecule drug	Parkinson’s disease	Enhanced therapeutic effect due to brain-specific drug delivery	Kunming mice blood	Co-incubation	[173]
miRNA	Genetic substances	Glioblastoma tumor	Provide diagnostic information	Glioblastoma cells	Transfection	[174]
miRNA	Genetic substances	Ischemia kidney injury	Protected kidney function and reduced kidney injury	Human cord blood endothelial colony-forming cells	Transfection	[175]
Signal regulatory protein α	Protein	Tumor	Enhanced phagocytosis of tumor cells	HEK293T cells	Transfection	[176]
Curcumin	Small molecule drug	Glioma	Improved targeted imaging and therapeutic effect	RAW 264.7 cells	Electroporation	[177]
Spherical nucleic acids	Genetic substances	Prostate cancer	3000-fold enhanced knockdown of miR-21	PC-3 cells	Naturally encased	[178]

## Data Availability

Not applicable.

## Abbreviations

Term	Full-Form
AC	Alternating current
AD	Alzheimer’s disease
AFM	Atomic Force Microscopy
BBB	Blood-brain barrier
BSE	Backscattered Electrons
CNS	Central nervous system
Cryo-TEM	Cryogenic transmission electron microscopy
CSF	Cerebrospinal fluid
CSP	Cysteine string protein
DEP	Dielectrophoretic
DLD	Deterministic lateral displacement
DLS	Dynamic Light Scattering
ELISA	Enzyme-linked Immunosorbent Assay
EOAD	Early-onset Alzheimer’s disease
ERT	Enzyme-replacement therapy
ESCRT	Endosomal sorting complex required for transport
EVs	Extracellular vesicles
FCM	Fluorescence Correlation Microscopy
GMP	Good manufacturing practices
GTPase	guanosine triphosphatase
HD	Huntington’s disease
KI	Knock in
LOAD	Late-onset Alzheimer’s disease
MHC	Major histocompatibility complex
miRNA	MicroRNA
MPS	Mononuclear phagocyte system
MVB	Multivesicular body
MVs	Microvesicles
NFTs	Neurofibrillary tangles
nPLEX	Nano plasmonic exosome
NSCLC	Non-Small-Cell Lung Cancer
NTA	Nanoparticle Tracking Analysis
PD	Parkinson’s disease
PEG	Polyethylene glycol
PM	Plasma membrane
SEC	Size exclusion chromatography
SEM	Scanning Electron Microscopy
siRNAs	Small interfering RNAs
SPR	Surface Plasmon Resonance
TEM	Transmission Electron Microscopy
TFF	Tangential flow filtration
TSC	Tuberous sclerosis complex
